# Prevalence and determinants of the place of delivery among reproductive age women in sub–Saharan Africa

**DOI:** 10.1371/journal.pone.0244875

**Published:** 2020-12-31

**Authors:** Kenneth Setorwu Adde, Kwamena Sekyi Dickson, Hubert Amu

**Affiliations:** 1 Department of Population and Health, University of Cape Coast, Cape Coast, Ghana; 2 Department of Population and Behavioural Sciences, School of Public Health, University of Health and Allied Sciences, Hohoe, Ghana; Center for Healthy Start Initiative, NIGERIA

## Abstract

**Introduction:**

Maternal mortality is an issue of global public health concern with over 300,000 women dying globally each year. In sub-Saharan Africa (SSA), these deaths mainly occur around childbirth and the first 24hours after delivery. The place of delivery is, therefore, important in reducing maternal deaths and accelerating progress towards attaining the 2030 sustainable development goals (SDGs) related to maternal health. In this study, we examined the prevalence and determinants of the place of delivery among reproductive age women in SSA.

**Materials and methods:**

This was a cross-sectional study among women in their reproductive age using data from the most recent demographic and health surveys of 28 SSA countries. Frequency, percentage, chi-square, and logistic regression were used in analysing the data. All analyses were done using STATA.

**Results:**

The overall prevalence of health facility delivery was 66%. This ranged from 23% in Chad to 94% in Gabon. More than half of the countries recorded a less than 70% prevalence of health facility delivery. The adjusted odds of health facility delivery were lowest in Chad. The probability of giving birth at a health facility also declined with increasing age but increased with the level of education and wealth status. Women from rural areas had a lower likelihood (AOR = 0.59, 95%CI = 0.57–0.61) of delivering at a health facility compared with urban women.

**Conclusions:**

Our findings point to the inability of many SSA countries to meet the SDG targets concerning reductions in maternal mortality and improving the health of reproductive age women. The findings thus justify the need for peer learning among SSA countries for the adaption and integration into local contexts, of interventions that have proven to be successful in improving health facility delivery among reproductive age women.

## Introduction

Maternal mortality is a global public health concern with approximately 810 maternal deaths occurring daily in 2017 [1]. About 94% of these deaths occur in low and lower-middle-income countries with more than 56% occurring in Sub-Saharan Africa (SSA) [2]. The high maternal mortality cases in SSA come at the backdrop of the agenda on Sustainable Development Goals (SDGs) set in the year 2015, with Goal Three seeking to promote the health of all reproductive age women [3, 4]. Target 3.1 specifically requires SSA countries to reduce the maternal mortality ratio (MMR) to less than 70 deaths per 100,000 live births by the year 2030 [4].

The high prevalence of maternal mortality in SSA has been attributed to the low patronage of antenatal care and skilled birth attendants (SBAs) [5, 6]. Maternal mortality cases mostly occur around childbirth and the first 24 hours after delivery [7–9]. Studies have, however, shown that childbirth at health facilities is one of the safest ways to prevent maternal morbidity and mortality [10–12]. The World Health Organization (WHO) has also encouraged at least four antenatal and postnatal care visits to help safeguard the health of pregnant women [13].

Although various SSA countries have put in measures to increase the utilization of health facilities for childbirth, it is still low in some countries [14]. Joseph et al. [14] observed from a cross-sectional survey in 80 low and middle -income countries that the utilization of health facilities for delivery was above 90% in 25 of the countries and below 40% in 11 countries. In Eritrea, only 16% of rural women utilize a health facility for delivery as compared to 73.2% in urban areas [15]. Despite the free maternal health services policy in Ghana and the free maternity service policy for all public hospitals in Kenya, only 59% of women utilize SBAs at a health facility in Ghana [16] while only 47.6% of deliveries occur at a health facility in Kenya [17]. In Tanzania, a study by Ngowi et al. [18] also observed that 78.6% of deliveries occurred at a health facility.

While there are a plethora of publications in various SSA countries on the place of delivery [11, 17–19], there appears to be a paucity in the literature at the SSA sub-regional level. The only study conducted at the SSA level was by Doctor et al. [20]. While the authors examined the place of delivery at the SSA level, they aggregated the countries into sub-regions and thereby failed to account for the variance in the prevalence and determinants of the place of delivery based on the respective countries included in their analysis. Consequently, their study failed to discuss country-specific policies that might influence the prevalence and determinants of the place of delivery. With the place of delivery being a key determinant in maternal mortality, we sought to examine the prevalence and key determinants of the place of delivery among women in specific sub–Saharan Africa countries using recent data. Accordingly, findings from this study will provide policymakers and the general populace with information that would help in reducing the high prevalence of maternal mortality and neonatal mortality contributed by SSA to the global burden.

## Materials and methods

### Source of data

The study made use of collective data from the most recent Demographic and Health Surveys (DHS) in 28 countries in SSA conducted between 2010 and 2018. The DHS is a nationwide study undertaken in five years intervals in several developing countries in Africa, parts of Asia and Latin America. The DHS follows consistent procedures in questionnaires design, sampling, data collection, data cleaning, coding, and analyses, which allows for comparability across countries [21, 22]. For this study, only women who had given birth in the five years preceding the survey were included, which is 167,763.

### Study variables

The main outcome variable was the place of delivery. The outcome variable was coded as 0 = ‘home’ and 1 = ‘‘health facility’ [19]. Fourteen explanatory variables were used namely: age, residence, women and partner’s level of education, wealth status, marital status, number of ANC visits, skilled ANC provider, getting medical help for self: money needed for treatment, distance to a health facility and getting permission to go, listening to the radio and watching television.

Age was classified in 5 –year grouping and categorized as 15–19 = 1, 20–24 = 2, 25–29 = 3, 30–34 = 4, 35–39 = 5, 40–44 = 6, and 45–49 = 7. Place of residence was captured as urban = 1 and rural = 2. Women and partner’s levels of education were captioned as no education = 1, primary = 2, secondary = 3, and higher education = 4. Wealth status was categorized as poorest = 1, poorer = 2, middle = 3, richer = 4, and richest = 5. Marital status was also categorized as married = 1, cohabitation = 2, widowed = 3, divorced = 4, and separated = 5. The number of Antenatal Care (ANC) visits was captured as less than four visits = 1 and four or more visits = 2. Skilled ANC provider was categorised as no = 0 and yes = 1. Getting medical help for self: money needed for treatment, distance to a health facility, and getting permission to go were captured as a big problem = 1 and not a big problem = 2. Listening to radio and watching television were recorded as not at all = 1, less than once a week = 2 and at least once a week = 3.

### Data analysis

Descriptive and inferential analyses were performed. The descriptive analysis reported results on background characteristics, country, and the prevalence of place of delivery. Two Inferential models were analysed using binary logistic regression. Model 1 explored the association between place of delivery and the country variable. Model 2 also explored the association between the outcome variables and all the explanatory variables. The results of Model 1 are presented as crude odds ratios (CORs) with 95% confidence intervals (CIs). Whereas Model 2 is presented as adjusted odds ratios (AOR) with 95% confidence intervals (CIs). Stata version 14 was used for the analysis. The multifaceted nature of the sampling structure of the DHS data was adjusted using the Stata Survey command ‘svyset v021 [pweight = wt], strata (v023)’, and the individual sample weight variable (v005).

### Ethical approval

Questionnaires and procedures for the surveys were reviewed and approved by the Ethics Committee of Opinion Research Corporation Macro International Inc and ICF Institutional Review Board (IRB). As nationally representative surveys, the DHS survey protocols for the various countries were also reviewed and approved by the ICF IRB and the relevant IRBs of the various countries. All data were completely anonymized, de identified, and/or aggregated before access and analysis. Detailed information on the ethical procedures observed by the DHS program can be accessed via http://goo.gl/ny8T6X. As we used secondary data for our analysis, we did not require further ethical approval from our named institutional bodies as the national level ethical clearance was sufficient for our analysis to be carried out.

## Results

### Background characteristics, country, and place of delivery

The overall prevalence of health facility delivery was 66% and this ranged from 23% in Chad to 94% in Gabon (Table 1). Women aged 20–24 years commonly delivered at the health facility (67.9%). Eight in ten women from urban areas delivered at a health facility. Women with higher education (94.6%), richest wealth status (90.6%), separated (77.9%), who had four or more ANC visits (76.9%) and received ANC from a skilled provider (72.9%) delivered at a health facility (Table 1). Women who listened to the radio almost every day (85.0%) and those who watched television almost every day (95.1%) had higher prevalence of health facility delivery. Women who did not have a big problem in terms of the distance to a health facility (72.9%), getting permission to go the health facility (67.5%), and getting the money needed for treatment (71.9%) delivered more at a health facility than those who had a big problem doing so (Table 1).

**Table 1:** Background characteristics and place of delivery among reproductive age women in SSA.

Variable	Place of Delivery			
	Home		Health Facility	
	Frequency N = 57,071	Percentage	Frequency N = 110,692	Percentage
**Age**				
15–19	3,491	35.3	6,408	64.7
20–24	11,265	32.1	23,771	67.9
25–29	14,306	32.4	29,915	67.5
30–34	11,937	33.3	23,906	66.7
35–39	9,029	35.1	16,668	64.9
40–44	5,026	39.9	7,565	60.1
45–49	2,017	45.1	2,459	54.9
**Place of residence**				
Rural	49,251	42.4	66,907	57.6
Urban	7,820	15.2	43,785	84.8
**Level of education**				
No education	35,378	53.3	30,999	46.7
Primary	16,175	27.9	41,882	72.1
Secondary	5,198	13.9	32,162	86.1
Higher	320	5.4	5,649	94.6
**Wealth status**				
Poorest	19,332	53.0	17,114	47.0
Poorer	15,468	43.3	20,298	56.7
Middle	12,073	35.5	21,948	64.5
Richer	7,434	23.1	24,763	76.9
Richest	2,764	9.4	26,569	90.6
**Marital status**				
Married	48,523	37.1	82,146	62.9
Cohabitation	6,410	21.9	22,901	78.1
Widowed	586	36.9	1,002	62.1
Divorced	558	31.5	1,215	68.5
Separated	974	22.1	3,428	77.9
**Partner’s educational level**				
No education	29,927	54.1	25,401	45.9
Primary	15,955	31.2	35,224	68.8
Secondary	9,628	19.8	38,976	80.2
Higher	1,561	12.3	11,091	87.7
**Number of ANC visits**				
Less than four	35,541	47.7	39,023	52.3
Four or more	21,530	23.1	71,669	76.9
**Skilled ANC provider**				
No	21,558	58.6	15,205	41.4
Yes	35,513	27.1	95,487	72.9
**Getting medical help for self: money needed for treatment**				
Big problem	35,199	39.1	54,740	60.9
Not a big problem	21,872	28.1	55,952	71.9
**Getting medical help for self: distance to health facility**				
Big problem	30,133	44.1	38,155	55.9
Not a big problem	26,938	27.1	72,537	72.9
**Getting medical help for self: getting permission to go**				
Big problem	12,498	41.0	17,961	59.0
Not a big problem	44,573	32.5	92,731	67.5
**Listening to radio**				
Not at all	30,322	42.6	40,912	57.4
Less than once a week	11,091	32.5	23,071	67.5
At least once a week	14,860	26.1	42,181	73.9
Almost every day	798	15.0	4,528	85.0
**Watching television**				
Not at all	45,980	42.3	63,614	57.7
Less than once a week	5,804	28.0	14,911	72.0
At least once a week	4,906	16.0	25,823	84.0
Almost every day	381	4.9	7,344	95.1
**Country**				
Benin, 2017–2018	1,104	14.4	6,553	85.6
Burundi, 2016–2017	852	11.3	6,669	88.7
Cameroon, 2011	1,090	36.1	1,933	63.9
Chad, 2014–2015	2,574	76.8	777	23.2
Comoros, 2012	361	22.1	1,276	77.9
Congo 2011–2012	347	6.9	4,658	93.1
Congo DR, 2013–2014	1,821	18.3	8,119	81.7
Cote d’Ivoire, 2011–2014	1,763	41.3	2,505	58.3
Ethiopia, 2011	4,750	68.2	2,219	31.8
Gabon, 2012	163	6.5	2,330	94.5
Ghana, 2014	954	26.0	2,707	74.0
Gambia, 2013	1,710	35.0	3,171	65.0
Guinea, 2012	2,352	48.0	2,551	52.0
Kenya, 2014	2,082	34.0	4,037	66.0
Lesotho, 2014–2015	476	21.3	1,756	78.7
Liberia, 2013	1,513	40.9	2,184	59.1
Malawi, 2015–2016	667	6.1	10,224	93.9
Mali, 2018	1,886	31.5	4,099	68.5
Mozambique, 2011	2,852	41.9	3,960	58.1
Namibia, 2013	213	14.5	1,253	85.5
Niger 2012	5,185	66.6	2,597	33.4
Nigeria, 2018	11,668	59.1	8,061	40.9
Rwanda, 2014–2015	421	8.0	4,880	92.0
Sierra Leone, 2013	2,934	45.3	3,544	54.7
Tanzania 2015–2016	2,012	36.1	3,568	63.9
Togo, 2013–2014	1,154	26.7	3,344	74.3
Uganda 2011	1,910	24.2	6,000	75.8
Zambia, 2013–2014	2,257	28.3	5,717	71.7
All Countries (total)	57,071	34.0	110,692	66.0

### Binary logistic regression on the determinants of the place of delivery among reproductive age women in SSA

The odds of giving birth in a health facility were highest in Malawi in the bivariable model (COR = 3.02, 95%CI = 2.72–3.35) which significantly declined to 1.88 (95%CI = 1.68–2.11) in the multivariable model. Conversely, Chad recorded the lowest likelihood of health facility delivery in the 1st model [COR = 0.05, 95%CI = 0.04–0.05] and this further declined marginally in Model II (AOR = 0.04, 95%CI = 0.03–0.04). We found that the probability of health facility delivery declined with increasing age. Women from rural areas had a lower likelihood (AOR = 0.59, 95%CI = 0.57–0.61) of delivering at a health facility compared to those from urban areas. The likelihood of delivering at the health facility increased with increasing the wealth status of women. For instance, women with the richest wealth status had a higher likelihood (AOR = 3.31, 95%CI = 3.10–3.53) of delivering at a health facility compared to those with poorest wealth status (Table 2). The probability of health facility delivery increased with the level of education. For instance, we observed that women with higher education (AOR = 4.45, 95%CI = 3.87–5.10) and women whose partners’ had higher education (AOR = 1.51, 95%CI = 1.40–1.62) were more likely to deliver in a health facility compared to those who had no education.

**Table 2:** Binary logistic regression of place of delivery among reproductive age women in SSA.

Variable	Crude Odds ratio (Confidence interval)	Adjusted odds ratio (Confidence interval)
**Country**		
Benin, 2017–2018	Ref	Ref
Burundi, 2016–2017	1.38***(1.26–1.52)	3.47***(3.11–3.86)
Cameroon, 2011	0.33***(0.30–0.36)	0.14***(0.13–0.16)
Chad, 2014–2015	0.05***(0.04–0.05)	0.04***(0.03–0.04)
Comoros, 2012	0.60***(0.53–0.69)	0.30***(0.26–0.35)
Congo 2011–2012	1.20***(1.09–1.33)	0.82**(0.72–0.92)
Congo DR, 2013–2014	0.55***(0.50–0.59)	0.36***(0.33–0.40)
Cote d’voire, 2011–2014	0.22***(0.20–0.24)	0.15***(0.13–0.16)
Ethiopia, 2011	0.10***(0.10–0.11)	0.10***(0.09–0.11)
Gabon, 2012	0.95(0.84–1.07)	0.33***(0.28–0.38)
Ghana, 2014	0.42***(0.38–0.46)	0.15***(0.14–0.17)
Gambia, 2013	0.26***(0.24–0.28)	0.10***(0.08–0.11)
Guinea, 2012	0.76***(0.16–0.19)	0.15***(0.14–0.17)
Kenya, 2014	0.25***(0.23–0.27)	0.09***(0.08–0.11)
Lesotho, 2014–2015	0.56***(0.50–0.63)	0.19***(0.17–0.22)
Liberia, 2013	0.21***(0.19–0.23)	0.10***(0.09–0.11)
Malawi, 2015–2016	3.02***(2.72–3.35)	1.88***(1.68–2.11)
Mali, 2018	0.33***(0.31–0.36)	0.30***(0.27–0.33)
Mozambique, 2011	0.32***(0.29–0.35)	0.16***(0.14–0.17)
Namibia, 2013	0.88*(0.76–1.01)	0.27***(0.23–0.32)
Niger 2012	0.12***(0.11–0.12)	0.08***(0.07–0.08)
Nigeria, 2018	0.12***(0.11–0.13)	0.05***(0.04–0.05)
Rwanda, 2014–2015	2.17***(1.92–2.45)	1.10(0.97–1.26)
Sierra Leone, 2013	0.23***(0.21–0.25)	0.11***(0.10–0.12)
Tanzania 2015–2016	0.31***(0.28–0.33)	0.49***(0.44–0.54)
Togo, 2013–2014	0.42***(0.38–0.46)	0.66***(60–74)
Uganda 2011	0.53***(0.49–0.57)	0.23***(0.21–0.25)
Zambia, 2013–2014	0.45***(0.42–0.49)	0.17***(0.16–0.19)
**Age**		
15–19		Ref
20–24		0.87**(0.82–0.93)
25–29		0.84***(0.79–0.88)
30–34		0.84***(0.79–0.89)
35–39		0.83***(0.78–0.88)
40–44		0.80***(0.74–0.85)
45–49		0.77***(0.70–0.84)
**Place of residence**		
Rural		0.59**(0.57–0.61)
Urban		Ref
**Level of education**		
No education		Ref
Primary		1.35***(1.30–1.40)
Secondary		2.21***(2.11–2.31)
Higher		4.45***(3.87–5.10)
**Wealth status**		
Poorest		Ref
Poorer		1.30***(1.26–1.35)
Middle		1.50***(1.44–1.56)
Richer		1.98***(1.90–2.07)
Richest		3.31***(3.10–3.53)
**Marital status**		
Married		Ref
Cohabitation		1.02(0.99–1.07)
Widowed		0.96(0.85–2.08)
Divorced		1.07(0.95–1.21)
Separated		1.09(1.00–1.18)
**Partner’s educational level**		
No education		Ref
Primary		1.24***(1.20–1.30)
Secondary		1.48***(1.42–1.54)
Higher		1.51***(1.40–1.62)
**Number of ANC visits**		
Less than four		Ref
Four or more		1.97***(1.91–2.02)
**Skilled ANC provider**		
No		Ref
Yes		4.13***(3.96–4.31)
**Getting medical help for self: money needed for treatment**		
Big problem		Ref
Not a big problem		1.02(0.99–1.05)
**Getting medical help for self: distance to a health facility**		
Big problem		Ref
Not a big problem		1.47***(1.43–1.52)
**Getting medical help for self: getting permission to go**		
Big problem		Ref
Not a big problem		1.02(0.98–1.06)
**Listening to radio**		
Not at all		Ref
Less than once a week		1.14***(1.10–1.18)
At least once a week		1.12***(1.09–1.16)
Almost every day		1.10**(1.00–1.21)
**Watching television**		
Not at all		Ref
Less than once a week		1.22***(1.17–1.28)
At least once a week		1.48***(1.41–1.54)
Almost every day		1.86***(1.64–2.10)

* p>0.10

** p>0.05

*** p>0.01

Women who had four or more ANC visits had a higher odd (AOR = 1.97, 95%CI = 1.91–2.02) of delivering at a health facility compared to those who had less than four ANC visits (Table 2). We found that women who received ANC from a skilled provider were more likely (AOR = 4.13, 95%CI = 3.96–4.31) to deliver at the health facility compared to those who did not receive ANC from a skilled provider. Women who did not have a big problem with the distance to health facility (AOR = 1.47, 95%CI = 1.43–1.52) had a higher likelihood to deliver at the health facility compared to those who had a big problem with distance to the health facility (Table 2). Furthermore, women who listened to radio almost every day (AOR = 1.10, 95%CI = 1.00–1.21) and those who watch television almost every day (AOR = 1.86, 95%CI = 1.64–2.10) had a higher likelihood to deliver at the health facility compared to those who did not listen to the radio at all and those who do not watch television at all (Table 2).

## Discussion

In this study, we examined the prevalence and determinants of the place of delivery among reproductive age women using data from the DHS of 28 SSA countries. The overall prevalence of health facility delivery was 66%. While the lowest prevalence of health facility delivery was recorded in Chad (23%), the highest was recorded in Gabon (94%). The determinants of the place of delivery were country, age, place of residence, level of education, wealth status, marital status, partner’s educational level, number of ANC visits, utilization of a skilled ANC provider during delivery, distance to a health facility, listening to the radio, and watching television.

We found that more than half of the 28 countries included in our analysis recorded a less than 70% prevalence of health facility delivery. These were Cameroon, Chad, Cote d’Ivoire, Ethiopia, Gambia, Guinea, Kenya, Liberia, Mali, Mozambique, Niger, Nigeria, Sierra Leone, and Tanzania. The implication is that these countries are far from achieving the SDG 3 of ensuring healthy lives and promoting the wellbeing of all reproductive age mothers [4]. Maternal mortality rates could, therefore, continue to be high in those SSA countries which then also defeats the SDG 3.1 target of reducing the maternal mortalities ration to below 70 deaths per 100,000 live births. Our findings point to the fact that interventions to improve health facility use and eventually reduce maternal mortality in the respective countries are probably either non-existent or are deficient.

Chad, for instance, has the highest MMR in SSA (856 per 100,000 live births) [23]. While the government has instituted an agenda to achieve the reduction of the country’s MMR to 500 per 100,000 live births by 2030 [24], this intervention has faced challenges emanating from limited infrastructure and health financing mechanisms available in the country [23]. It was, therefore, not surprising that Chad recorded the lowest probability of health facility delivery among the 28 countries included in our analysis. Ethiopia also has one of the highest MMR in SSA (401 per 100,000 live births) [25]. This is despite the implementation of maternal health interventions which have included the roll-out of basic obstetric care [26], and the strengthening of existing institutions in rural areas, improving the quality and capacity of work at health facilities and increasing referrals to hospitals through the use of health extension workers [27].

An important intervention common in many of the countries with a high health facility delivery in our study is the implementation of a unified social health insurance schemes which provide health coverage for the general populace with special provisions for reproductive age women, particularly targeting childbirth. Gabon, for instance, implemented the Caisse Nationale d’Assurance Maladie et de Garantie Sociale (CNAMGS) in 2008 which covers all maternal health services in the country and greatly reduces the cost of childbirth and the skilled delivery process [28]. Democratic Republic of Congo and Ghana also have similar successful health insurance interventions [29, 30] which could be credited for the high health facility delivery we observed in those countries.

Aside from social health insurance, demand-side interventions for maternal care focused on community-based mobilizations, have proven successful in other countries which recorded high health facility delivery in our study. In Malawi, for instance, this involved the use of trained facilitators who led varied forms of discussion groups to improve knowledge of health problems when eventually resulted in increased health facility delivery [31]. This explains why Malawi had the highest odds of health facility delivery in our study. In Zambia, this was in the form of a community-based intervention called the Safe Motherhood Action Groups (SMAGs) made up of women and men [32]. It is prudent for peer learning by the SSA countries where countries with lower health facility delivery found in our study would adapt interventions that have been successful in countries with higher health facility delivery.

We found that the odds of choosing a health facility as the place of delivery declined with age among reproductive age women in SSA. This points to women’s perception of their susceptibility to maternal health complications by age especially during childbirth and how these age-specific perceptions influence the seriousness they attach to skilled maternal health care utilisation and actual health facility use for childbirth. Younger women who are probably giving birth for the first time, are naturally at higher risks of maternal complications than older ones who are usually multiparous women [33]. As such, they tend to access health facility delivery more than older women to receive the best clinical care possible and to avoid such complications [34]. The older women on the other hand, with reduced possibilities of birth complications due to being multiparous, usually prefer home delivery using Traditional Birth Attendants (TBAs) for delivery as TBAs are considered as being more friendly and caring compared to SBAs [34]. It was, therefore, not surprising in our study that the highest prevalence of home delivery was recorded among women in their last reproductive years [45–49].

In our study, the prevalence and probability of choosing a health facility as the place of delivery were higher among urban women in their reproductive years than those from rural areas. In most SSA countries, there are vast disparities between rural and urban areas in terms of the siting of health facilities including those providing skilled delivery services to the advantage of urban areas [35–37] and this reflects the higher health facility utilisation found in our study among urban women. Closely related to the rural-urban disparities was the fact that in our study, women who considered the distance to a health facility as a big problem had a lower probability of utilising health facilities for delivery. Thus, as women in rural areas are disadvantaged in terms of the citing of health facilities, they probably have to travel long distances to access skilled delivery services in the urban areas which experience a multiplicity of these facilities in the sub-region [38, 39]. The distance, thus, becomes a big problem that deters them from utilising health facilities for delivery as the roads from rural areas are usually in deplorable conditions, in addition to the high cost of transportation fares to the urban areas which most of the women find difficult to afford.

The level of education was an important determinant of the place of delivery in our study. We found that the probability of giving birth in a health facility increased by increasing the level of education among the reproductive age women and their partners respectively. The findings are indicative of the essential role that formal education plays in women’s reproductive health decision making in SSA [40, 41]. Formal education, for instance, empowers women through the provision of essential information needed to make informed reproductive health decisions which in the case of our study, was health facility delivery, to safeguard their health and that of their babies. Education also provides women with some autonomy in decision making regarding their health [41, 42]. In SSA, however, this autonomy becomes weakened for women in union. This is because male partners play a key role in the reproductive health decision making of the women as they are revered as the family heads who take the final household decisions including those affecting childbirth [43–46]. The fact that the odds of utilising health facility for delivery in our study increased with a partner’s level of education, however, implies that the more educated a woman’s partner is, the more likely they were to support them in their reproductive health decision making.

In our study, the prevalence and probability of giving birth at a health facility as the place of delivery increased with increasing the wealth status of the women. In SSA, financial constraints in the access and utilisation of health services are highly prevalent and preclude many people especially the poor from utilising the health services. This was evident in our finding where the prevalence and odds of utilising health facilities for delivery were higher for women who did not consider money needed for treatment as a big problem, though not statistically significant. While interventions to ensure the financial health protection in SSA have been largely pro-poor, the majority of people who benefit from such interventions which include health insurance, are those in highly wealth quintiles, leaving out the poor [47, 48]. Policies to improve the health facility utilisation in SSA countries have to, therefore, not only be designed as pro-poor, but also implemented with a focus on meeting the needs of the poor who need them most.

We realised that watching television and listening to the radio were important determinants of the place of delivery among reproductive age women in favour of health facility delivery. For instance, the more frequent women watched television, the more likely it was for them to give birth at a health facility. The findings reflect the increasing role of the media in positively influencing health-seeking behaviour in SSA [49–51]. With the advent of electronic media and the proliferation of media outlets [52] airing various health-related programmes including those related to reproductive health, women who frequently watch/listen to such programmes become better informed to seek skilled reproductive healthcare than those who do not. There is, therefore, the need for more health-related content on radio and television stations in the sub-region which would further increase the choice of health facility for delivery among women of reproductive age.

### Strengths and limitations

Our study was the first attempt at understanding the multi-country level prevalence and determinants of the place of delivery in SSA while focusing on the various countries included in the analysis. It, therefore, contributes immensely not only to the literature on place of delivery in the sub-region but specifically establishes the variations based on the individual countries. Our use of DHS data ensured that the data were representative of the various countries included in our analysis. Our use of regression analysis also ensured that we effectively examined the determinants of the place of delivery among the women. A major limitation of the study, however, was the cross-sectional nature of the data used which made it difficult to measure causality.

## Conclusions

Our findings point to the inability of many SSA countries to meet the SDG targets concerning reductions in maternal mortality and improving the health of reproductive age women. The findings thus justify the need for peer learning among SSA countries for the adaption and integration into local contexts, of interventions that have proven to be successful in improving health facility delivery among reproductive age women. Effective implementation of harmonized social health insurance schemes and community-based mobilisation for maternal healthcare are some of these interventions. In the adoption of these interventions, special considerations could be given to the poor, older reproductive age women, rural women, and those without any formal education.

## Abbreviations

ANC: Antenatal Care
AOR: Adjusted Odds Ratio
COR: Crude Odds Ratio
DHS: Demographic and Health Survey
MMR: Maternal Mortality Ratio
SBAs: Skilled Birth Attendants
SDG: Sustainable Development Goal
SSA: Sub-Saharan Africa
TBAs: Traditional Birth Attendants
WHO: World Health Organisation
